# Multi-sensor microclimate and tree canopy dataset from a linden tree for green infrastructure validation

**DOI:** 10.1038/s41597-026-07536-1

**Published:** 2026-06-01

**Authors:** Peer Schöneberger, Tim Sinsel, Helge Simon, Michael Bruse

**Affiliations:** Department of Geography, Johannes Gutenberg University Mainz, 55099 Mainz, Germany

## Abstract

Validating microclimate simulation models requires accurate *in-situ* measurements across multiple spatial locations and environmental parameters. This dataset provides a detailed collection of microclimate observations gathered around a linden tree (*Tilia cordata*) located in Grünstadt, Rhineland-Palatinate, Germany, over a three-day period in June 2025. It includes high-resolution measurements of air temperature, humidity, barometric pressure, shortwave and longwave radiation, illuminance, leaf surface temperatures through thermal imaging, tree-sap flow, and a three-dimensional point cloud of the canopy structure. The data were collected using a combination of professional-grade instruments and low-cost microcontroller-based sensors, allowing for an assessment of affordable alternatives in urban microclimate monitoring. Validation against professional-grade reference instruments demonstrated an R^2^ > 0.99 for air temperature and strong agreement for radiative fluxes (R^2^ = 0.9888, RMSE = 35.7 W m^−2^), confirming the viability of these affordable alternatives. All data are available in standardized formats (CSV for time-series, LAS for point clouds, JPG for images) with comprehensive metadata documentation. This dataset supports research in sensor calibration, microclimate model validation, and the feasibility of high-resolution spatial monitoring using cost-effective sensor networks in urban vegetation studies.

## Background & Summary

Microclimatic validation through *in-situ* measurement networks is essential for developing and testing microclimate simulation models^1,2^. Professional meteorological instrumentation and integrated data acquisition systems, however, represent significant financial barriers to many research institutions. When high spatial resolution data are required, such as distributed radiation measurements beneath tree canopies or spatially resolved leaf temperature distributions, the number of sensors needed quickly exceeds typical institutional procurement capacities. Additionally, commercial integrated systems often exhibit rigid architectures that cannot accommodate diverse sensor types or custom logging intervals. In contrast, programmable microcontroller platforms offer the necessary flexibility to overcome these limitations. These practical considerations motivated the development of a customized, low-cost sensing system, which was deployed *in-situ* to systematically assess its performance and potential as a viable alternative for distributed microclimate measurements^3–5^.

Efforts to map urban environments generally fall into two categories: city-scale networks and hyperlocal mobile sensing. The former utilizes high-density temperature networks and national-scale model-observation products to resolve broad meteorological fields at kilometer resolution^6^. In contrast, the latter leverages mobile and low-cost platforms to obtain hyperlocal data within complex urban geometries, primarily focusing on air quality and near-surface microclimates^7^. However, these low-cost distributed systems often struggle with data quality, creating a need for the high-fidelity, multi-sensor validation dataset required to evaluate green infrastructure.

The present contribution evaluates the viability of low-cost, open-source sensing platforms as alternatives to professional-grade meteorological instrumentation. The underlying research question focuses on whether these affordable systems can maintain the level of accuracy required for detailed microclimate model validation. By characterizing measurement uncertainties under field conditions, this study demonstrates how resource-limited research groups might utilize distributed sensing to achieve high-resolution spatial sampling.

Urban green infrastructure, particularly street trees and park vegetation, provides multiple ecosystem services including cooling effects through shading and transpiration^8,9^. Microclimate simulation software such as ENVI-met enables researchers to explore how vegetation geometry and physiological characteristics influence local thermal environments^10,11^. However, validating whether simulated tree canopy properties produce accurate microclimate predictions requires detailed field measurements capturing the spatial heterogeneity of the radiation environment, three-dimensional canopy structure, and leaf-level energy exchange processes. Several open datasets provide urban-scale information on tree canopy cover and urban form that support microclimate modelling and assessment of urban heat islands, but they typically lack co-located measurements of sub-canopy radiation and leaf-level processes^12,13^. Canopy-scale datasets from forest settings have demonstrated how tree species differ in their ability to regulate canopy temperature and microclimate during drought and heat events, based on combined measurements of canopy temperature, radiation and local meteorological forcing^14–16^.

Moving toward a more granular understanding of urban green infrastructure, this study focuses on the sub-canopy dynamics of a common street tree, represented here by a single mature linden tree (*Tilia cordata*), a ubiquitous urban species in central Europe and representative of typical planted vegetation in urban settings^17^. A systematic spatial grid was employed to capture the heterogeneous radiation environment beneath the canopy, thereby supporting validation of how microclimate models represent tree shade dynamics across space and time. The present measurements extend such canopy microclimate perspectives to a typical urban street tree exposed to high radiant loads, while adding detailed sub-canopy radiation and physiological measurements. The dataset should thus be regarded as a detailed case study and benchmark for a mature *Tilia cordata* tree under high-radiation conditions, rather than a multi-species or site-wide climatology study.

The measurement campaign integrated multiple complementary data streams: terrestrial LiDAR scans characterizing canopy geometry, distributed radiation sensors beneath the canopy, leaf surface temperature measurements, sap flow measurements, and environmental variables including air temperature, humidity, and barometric pressure. This integrated approach provides a complete characterization of tree physiological response and microclimate dynamics during a hot weather period, capturing conditions representative of thermal stress events.

The dataset supports multiple reuse pathways within the research community. First, researchers can evaluate the accuracy and measurement stability of low-cost environmental sensors by comparing simultaneous measurements from multiple sensor types and data logging systems operating under known environmental conditions. This intercomparison capability advances understanding of sensor performance and contributes to methodological development in low-cost sensor networks. Second, the combination of detailed canopy structure data derived from terrestrial LiDAR with comprehensive environmental measurements enables validation of microclimate simulation models and assessment of how accurately canopy geometric properties are translated into predicted radiation and temperature fields. Third, the integrated measurements of leaf temperature, sap flow, and radiation absorption provide data relevant to studies of urban tree thermal stress, water relations, and physiological responses to environmental forcing.

Beyond sensor and model applications, the documented calibration procedures and uncertainty quantification approaches represent reusable methodologies applicable to other distributed sensing campaigns, particularly for research groups establishing high-resolution monitoring networks with limited budgets. The dataset includes detailed metadata and file structure documentation relevant to establishing community standards for archiving complex, multi-instrument field campaigns^18^. Comparable urban environmental data products have emphasized documentation of calibration strategies and uncertainty assessment, for example in multi-city greenhouse-gas monitoring networks and national-scale urban meteorology datasets that follow FAIR data principles and provide error estimates^6^. Similarly, recent low-cost sensing initiatives have demonstrated the value of publishing not only high-resolution measurements but also standardized calibration and validation workflows as part of the dataset descriptor^7^. These documentation practices contribute to broader efforts in advancing open science and reproducible research by providing a transparent example of data management and quality assurance procedures applicable across distributed sensing applications. The dataset is intended to support methodological development in sensor validation, microclimate model evaluation, and the practical implementation of budget-conscious monitoring strategies in environmental research. By bridging the gap between three-dimensional structural documentation and high-resolution, co-located measurements of leaf physiology and sub-canopy microclimate, this dataset offers a uniquely integrated characterization of tree-atmosphere interactions that distinguishes it from existing single-parameter or large-scale urban meteorological networks.

## Methods

The following section outlines the methodological framework of the measurement campaign and the resulting dataset. It first introduces the experimental setup and spatial configuration used to capture the microclimate variability around a representative urban tree, followed by detailed descriptions of the instruments and data acquisition for atmospheric parameters, radiation, leaf surface temperature, sky conditions, sap flow, the three-dimensional canopy structure and a cost analysis of the used measurement instruments. Together, these complementary measurements form an integrated dataset designed to quantify energy exchange processes at the interface between trees and the atmosphere.

### Experimental design

The measurement campaign was designed to comprehensively characterize the microclimate environment surrounding a mature urban linden tree, a species representative of common tree plantings across central European cities. Emphasis was placed on capturing spatial heterogeneity of radiation receipt and leaf-level thermal properties to develop and validate a scalable methodology applicable beyond this exemplar. To resolve sub-canopy variability, a regular orthogonal grid centered on the tree stem was established, aligned with the cardinal directions (north, south, east, west), with 2 m horizontal spacing between measurement stations. This configuration established a 14 m × 14 m measurement domain comprising 49 discrete sampling locations, of which 48 positions represented distributed illuminance sensors at ground level (~10 cm above ground) for high-resolution mapping of visible light beneath the canopy. In addition to this ground-based sensor array, co-located measurement systems recorded atmospheric parameters (air temperature, humidity, and pressure), broadband radiation in the immediate vicinity of the tree, leaf surface temperatures from multiple viewing angles, sap flow within the stem, and three-dimensional canopy structure using terrestrial LiDAR, as detailed in the following sections. A comprehensive summary of all technical specifications, manufacturers, and sensor accuracies is consolidated in Table 1.

**Table 1:** Technical specifications and manufacturers of the instrumentation used for the measurement campaign.

Parameter	Device / Sensor Model	Manufacturer	Key Specifications / Accuracy
Air Temperature & Relative Humidity (Reference)	CS215-L	Campbell Scientific Inc.	±0.4 °C /±2% accuracy
Air Temperature & Relative Humidity (Low-Cost)	AHT10	Aosong Electronic Co. Ltd.	±0.3 °C /±2% accuracy
Barometric Pressure	BMP280	Bosch Sensortec GmbH	±1 hPa accuracy
Net Radiation	SN-500	Apogee Instruments Inc.	4-component (SWin, SWout, LWin, LWout); ±5% daily totals accuracy
Illuminance	BH1750FVI	ROHM Semiconductor Co. Ltd.	400–700 nm range
Fixed Thermal Imaging	MLX90640	Melexis N.V.	32 × 24 pixel; 18-bit; ±2 °C accuracy
Mobile Thermal Imaging	E6 Pro	FLIR Systems Inc.	240 × 180 pixel; ±2 °C accuracy
Sap Flow	TDP SF-G	Ecomatik GmbH	Thermal dissipation; 33 mm length
Sky Imagery	Raspberry Pi 5 Cam	Raspberry Pi Foundation	120° field-of-view fisheye lens
Canopy Structure	GLS-1500	Topcon Corporation	Terrestrial Laser Scanner (LiDAR)
Microcontroller	ESP32	Espressif Systems Co. Ltd.	MicroSD and RTC integration
Data Logger	CR1000	Campbell Scientific Inc.	13-bit ADC

### Site selection and tree characteristics

**Fig. 1:**
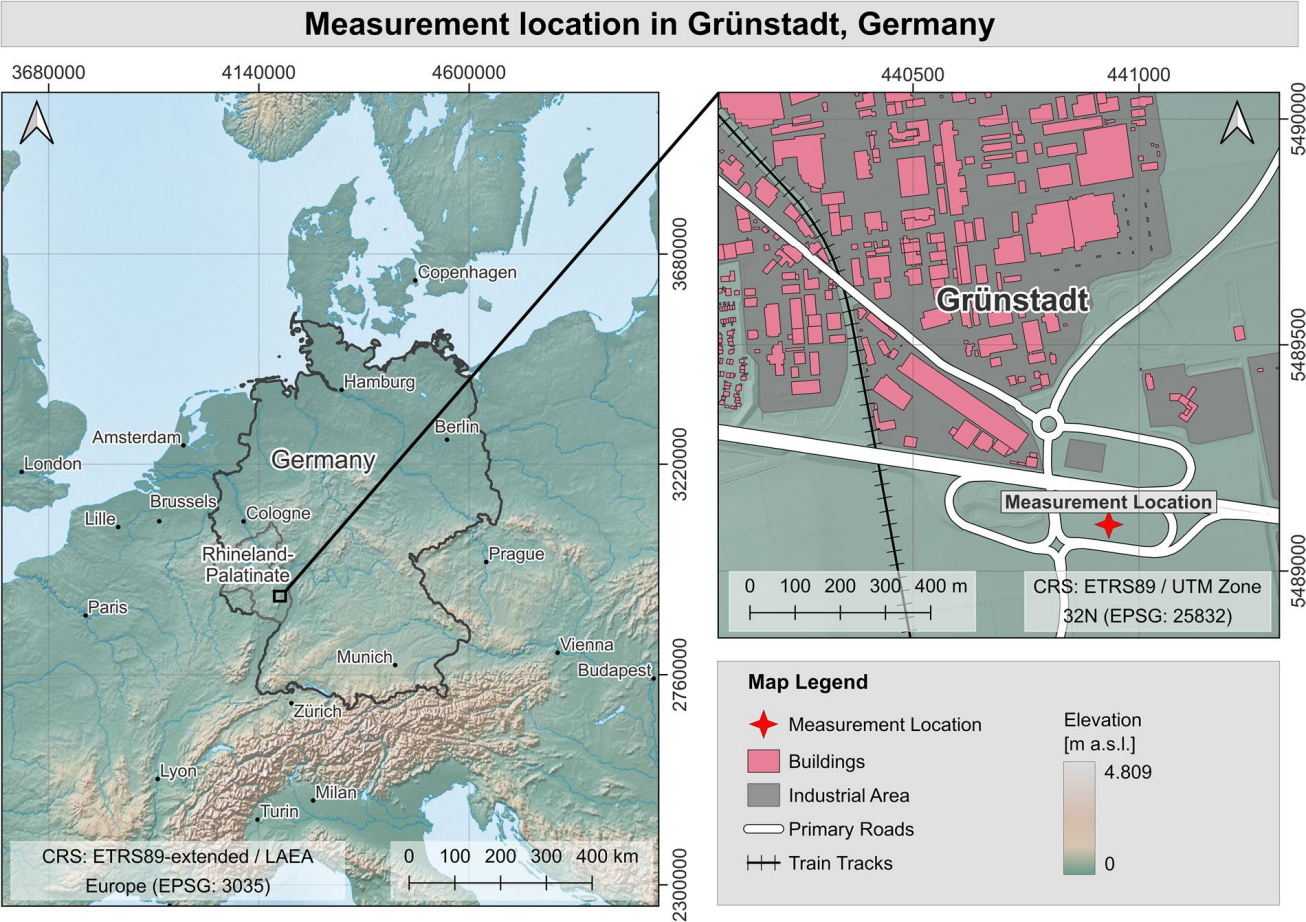
Measurement location in Grünstadt, Rhineland-Palatinate, Germany.

The measurement site was located in a green corridor bordering a highway interchange near Grünstadt, Rhineland-Palatinate, Germany (49° 33′ 05.24″ N, 8° 11′ 00.50″ E, elevation 165 m above sea level) (Fig. 1). The site was selected based on multiple criteria: accessibility for instrument installation and maintenance, minimal vandalism risk due to semi-isolated location within a roadside vegetation strip, absence of disease or physical damage to the target tree, and the opportunity to measure a specimen of *Tilia cordata* representative of urban plantings. At the time of the measurement the surveyed tree was 30 years old with a stem diameter at breast height (DBH) of 31 cm and a total height of 9.64 m. Canopy lateral extent measured 10.61 m in the north-south direction and 10.93 m in the east-west direction. Leaf clusters extended to ground heights between 1.0 and 1.2 m at the canopy margins.

Through terrestrial LiDAR scanning the tree’s three-dimensional structure was documented (see Three-Dimensional Canopy Structure section). Quantitative characterization from the point cloud indicated a Leaf Area Index (LAI) of 4.4 m^2^ m^−2^, consistent with literature values for *Tilia cordata* in mature specimens^19,20^. Leaf Area Density (LAD) manually derived from a dense leaf cluster on the tree’s north side measured 2.04 m^2^ m^−3^. These structural metrics correspond to a relatively dense, broad-crowned deciduous tree, so the microclimate and radiation patterns documented in this dataset are most directly transferable to species and plantings with comparable LAI and crown architecture.

**Fig. 2:**
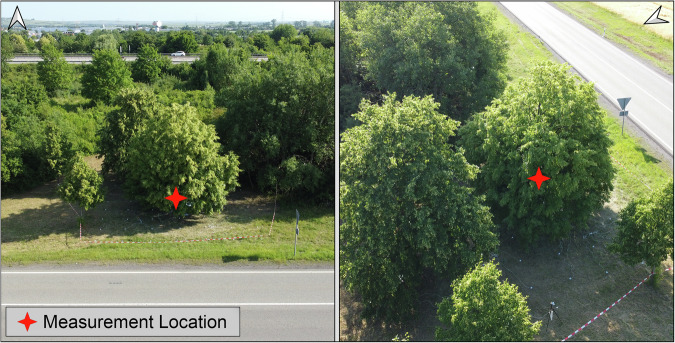
Aerial and front view of the measurement area.

The measurement location was characterized by the presence of additional tree individuals: a mature *Tilia cordata* specimen positioned immediately to the north, a large deciduous tree to the east, and a juvenile tree to the west of the study tree (Fig. 2). The proximity of these trees to the measurement domain potentially influenced radiation receipt at the measurement grid, particularly at the northern, eastern and western margins. This feature is documented in the dataset and should be considered during data analysis and model validation comparisons^18^.

### Measurement period

Data collection spanned 2025-06-11 19:05 to 2025-06-14 19:05 UTC + 1 (72 hours), deliberately capturing intensive and largely clear-sky conditions essential for quantifying radiation-driven heat stress dynamics beneath urban tree canopies. This period represented peak summer conditions with high solar forcing, minimal cloud interference, and stable atmospheric stability, namely critical boundary conditions for microclimate model validation under thermal stress scenarios. The duration was constrained by the requirement for uninterrupted clear-sky weather to ensure consistent radiation and leaf temperature measurements, combined with an abrupt thunderstorm termination in the afternoon on June 14. Extension was precluded by impending instrument retrieval logistics and seasonal field campaign scheduling. Consequently, the temporal coverage of the dataset is intentionally limited to a single, uninterrupted 72-hour clear-sky episode in mid-June. This design provides well-defined boundary conditions for model validation under high-radiation forcing, but it does not capture cloudy or rainy conditions, synoptic variability, or seasonal changes in background climate and canopy state.

### Atmospheric parameter measurement

Air temperature and relative humidity were measured using two independent measurement systems deployed simultaneously within a radiation shield mounted at 2 m height above ground level. The professional-grade reference system employed a CS215-L combined sensor (Campbell Scientific Inc., Logan, Utah, USA) logged by a CR1000 datalogger (Campbell Scientific Inc.), offering temperature accuracy of ±0.4 °C with 0.01 °C resolution and relative humidity accuracy of ±2% with 0.03% resolution^21^. This reference system was sampled at 5-second intervals throughout the measurement period. In parallel, a low-cost microcontroller-based system recorded simultaneous measurements using an AHT10 sensor (Aosong Electronic Co. Ltd., Guangzhou, China) connected to an ESP32 microcontroller (Espressif Systems Co. Ltd., Shanghai, China) with real-time clock functionality. The AHT10 sensor specifications included a temperature accuracy of ±0.3 °C at a resolution of 0.01 °C and a relative humidity accuracy of ±2% at 0.024% resolution, with data acquisition occurring at 20-second intervals^22^. Both sensors were housed in the same radiation shield and data from the low-cost system were transmitted to a microSD card with automatic timestamp association. All timestamps were recorded by a real-time clock module (DS3231), ensuring temporal synchronization with the temperature and humidity measurements.

Barometric pressure measurement was conducted at 2 m height using a BMP280 sensor (Bosch Sensortec GmbH, Gerlingen, Germany) connected to an ESP32 microcontroller with data logged continuously to a microSD card. The pressure sensor achieved an accuracy of ±1 hPa with 0.16 Pa resolution and operated on a 20-second sampling interval^23^.

### Radiation measurement

Radiation measurements encompassed both broadband spectral components and high-resolution spatial mapping of visible light. A SN-500 Net Radiometer (Apogee Instruments Inc., Logan, Utah, USA) was deployed at 1.5 m height above ground on an open area immediately adjacent to the measurement tree, positioned to ensure no shading from the canopy. This instrument measured net shortwave and longwave radiation components with calibration uncertainty for daily totals of ±5%^24^. Data were sampled at 5-second intervals and logged by a CR1000 datalogger (Campbell Scientific Inc.), yielding output variables including incoming shortwave radiation (SWin), outgoing shortwave radiation (SWout), incoming longwave radiation (LWin), outgoing longwave radiation (LWout), and derived net radiation components (SWnet, LWnet).

To achieve high spatial resolution mapping of radiation heterogeneity beneath the canopy, illuminance was measured using 49 BH1750FVI ambient light sensors (ROHM Semiconductor Co. Ltd., Kyoto, Japan) that respond to wavelengths in the 400–700 nm range^25^. According to the datasheet, at 1000 lux the ratio between the sensor output and the actual illuminance is specified to typically be 1.20, with a possible range from 0.96 to 1.44^25^. Each sensor was fitted with a hemispherical diffuser geometrically similar in design to the SN-500 radiometer to ensure comparable angular response characteristics across all measurement positions. One BH1750FVI sensor was positioned immediately adjacent to the SN-500 radiometer at 1.5 m height to provide a calibration reference and establish the empirical relationship between photometric and radiometric units. Data from this reference sensor were acquired via the I²C interface with 20-second sampling using an ESP32 microcontroller, with timestamps synchronized to a real-time clock module.

**Fig. 3:**
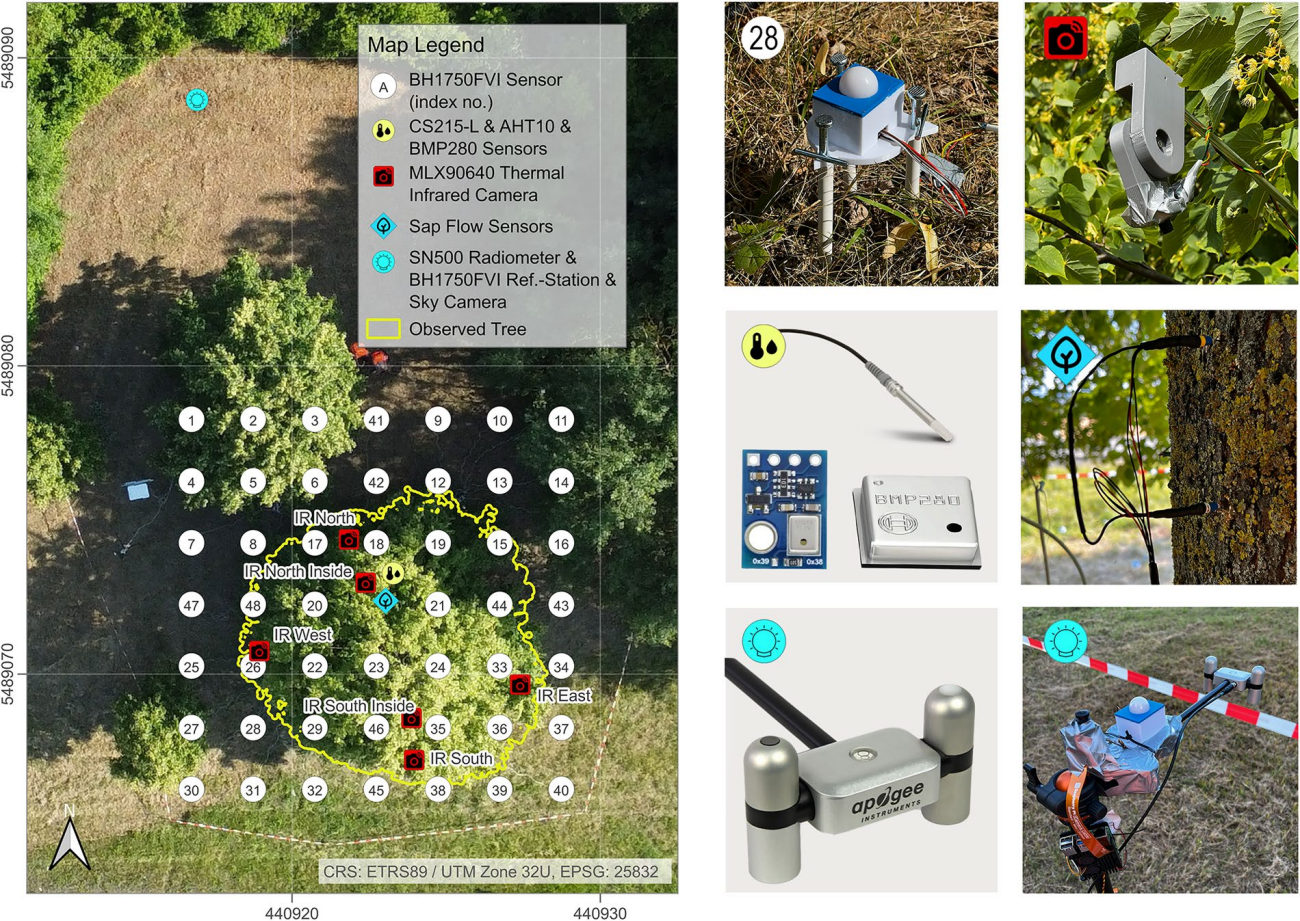
Overview of the sensors installed in the measurement area.

The remaining 48 BH1750FVI sensors were deployed across the 14 m × 14 m measurement grid at ground level (~10 cm above ground) to characterize spatial variation in visible light receipt beneath the canopy (Fig. 3). To manage the I²C addressing limitations inherent in the BH1750FVI protocol, six I²C multiplexers were implemented in a hierarchical architecture, with each multiplexer managing eight sensors. This configuration enabled simultaneous reading of all 48 sensors with a single ESP32 microcontroller while maintaining temporal synchronization across the entire sensor array. The BH1750FVI was operated at reduced resolution to accommodate the high illuminance values from direct solar radiation. This operational setting resulted in a measurement step size of approximately 7.4 lux per count, representing an intentional trade-off between dynamic range and sensitivity. Raw data were initially stored in lux units and subsequently converted to radiometric units (W m^−2^) using a quadratic regression derived from simultaneous measurements with the reference radiometer (see Technical Validation).

### Leaf surface temperature measurement

Leaf surface temperature monitoring was accomplished through two complementary approaches providing different temporal resolution and spatial coverage. Six thermal infrared cameras based on the MLX90640 radiometric sensor (Melexis N.V., Ypres, Belgium) were deployed for continuous fixed-position measurements throughout the campaign. Each sensor unit measured a 32 × 24 pixel thermal image with 16:9 field-of-view (55° × 35°) and operated with an accuracy of ±2 °C and 18-bit thermal resolution^26^. The sensors were mounted at approximately 3 m height on rigid aluminum fixtures oriented toward the outer canopy foliage in the four cardinal directions (north, south, east, west) and at two internal locations beneath the canopy (north and south) (Fig. 3). This spatial arrangement captured thermal variation of sunlit outer leaves and simultaneously provided comparison points within the shaded interior canopy environment.

Each MLX90640 unit was connected to an independent ESP32 microcontroller configured to handle I²C communication, provide real-time clock functionality for timestamp assignment, and manage microSD card data logging. All thermal cameras operated on a 20-second measurement interval, recording the complete set of 768 thermal pixels per camera at each time step. Emissivity was set to 1.0 for all measurements (see Technical Validation for calibration procedures and accuracy considerations).

To supplement the fixed thermal imaging with independent validation data, a FLIR E6 Pro thermal camera (FLIR Systems Inc., Wilsonville, Oregon, USA) with 240 × 180 pixel resolution was deployed for mobile measurements on 2025-06-12 between 06:20 and 19:20 UTC + 1. Measurements were conducted at approximately 15-minute intervals, systematically targeting identical leaf surfaces and viewing angles as the fixed MLX90640 cameras to enable direct intercomparison. Image metadata recorded measurement location (cardinal direction, canopy position) within each JPG file. Unlike the fixed cameras that provide pixel-by-pixel thermal data, the FLIR camera provided only aggregated metrics per image including center-spot temperature, minimum, and maximum values across the field-of-view. The measurement accuracy of this instrument is ±2 °C, with the emissivity set to 1.0^27^.

### Sky conditions and cloud cover documentation

Hemispherical sky imagery was acquired continuously throughout the measurement period using a Raspberry Pi 5 (Raspberry Pi Foundation, Cambridge, United Kingdom) equipped with a 120° field-of-view fisheye lens camera and a custom image acquisition script. Images were captured at 15-second intervals and stored as JPG files with timestamp metadata. This imagery enables quantitative characterization of cloud cover fraction and atmospheric conditions at each moment in the time series and supports post-hoc analyses of how aerosol optical depth or atmospheric transparency influenced radiation measurements.

### Sap flow measurement and transpiration estimation

The tree transpiration was quantified using the thermal dissipation probe (TDP) method of Granier^28^, which provides continuous measurements of radial sap velocity within the sapwood. A pair of TDP sensors (Type SF-G, Ecomatik GmbH, Dachau, Germany) with 33 mm length and 1.5 mm diameter were inserted to 23 mm depth into the sapwood on the north-facing stem at 1.75 m height. Sensors were installed after careful removal of bark in aluminum tubing to prevent thermal coupling between the measurement elements. The trunk was subsequently wrapped in reflective and insulating material to minimize solar radiation, as well as the resulting ambient air temperature and its impact on the measurement. The upper probe was continuously heated at 84 mA with a total heating power of 0.2 W using a CCS heating controller (Ecomatik GmbH), while the lower, unheated probe recorded the sapwood reference temperature. Temperature difference (*ΔT*) between the two probes was logged every 5 seconds as a differential voltage by a CR1000 datalogger (Campbell Scientific Inc.). Measurement specifications derived from the copper-constantan thermocouples output were used to calculate representative temperatures using a lookup table for Type T thermocouples based on NIST ITS-90 standards.

**Fig. 4:**
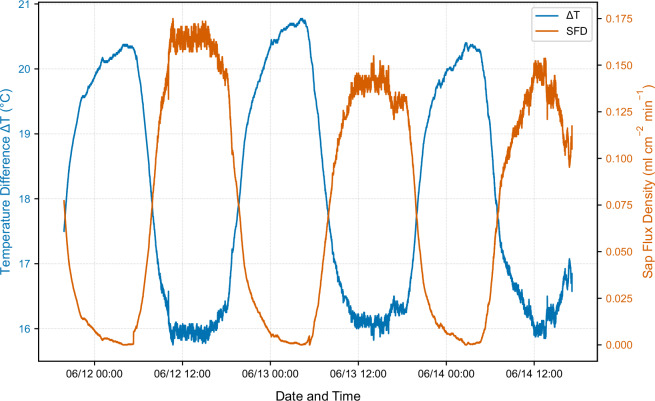
Visualization of ΔT and sap flux density during the measurement period.

Raw *ΔT* measurements were converted to sap flux density (U) [ml cm^−2^ min^−1^] using the Granier equation (Eq. 1):

1 $$U=0.714\cdot {\left(\frac{{\triangle T}_{\max }-\Delta T}{\Delta T}\right)}^{1.231}$$

where $\Delta {T}_{\max }$ represents the maximum temperature difference recorded during nighttime hours (when sap flow is negligible and *ΔT* is maximal)^28,29^. Daily maximum values were recalculated each day. The sap flow of the whole tree (F) [ml min^−1^] was subsequently calculated as (Eq. 2):

2 $$F=U\cdot {A}_{s}$$

where ${A}_{s}$ is the sapwood area [cm²]^28,29^ (Fig. 4).

The sapwood area was estimated from the measured DBH (31.2 cm) using the species-specific power function for *Tilia cordata* (Eq. 3)^30^.

3 $${A}_{s}=2.635\cdot {{\rm{DBH}}}^{1.561}$$

This calculation yielded an estimated sapwood area of 566.44 cm², corresponding to 73.9% of the stem cross-sectional area, consistent with independent measurements and literature values for this species^29,30^.

### Three-Dimensional Canopy Structure: LiDAR Acquisition and Processing

A three-dimensional characterization of the canopy structure was accomplished through terrestrial laser scanning using a Topcon GLS-1500 3D laser scanner (Topcon Corporation, Tokyo, Japan). Three independent scan positions were employed to ensure comprehensive coverage of the entire canopy structure while managing the scanning time and environmental conditions. The three scan positions were strategically located around the tree to provide complete angular coverage with adequate overlap between adjacent scans to ensure seamless point cloud merging. The resulting combined point cloud from these three acquisitions contained approximately 29 million points from the raw unfiltered scans.

Data processing was conducted using the Topcon ScanMaster software (Topcon Corporation) to systematically identify and remove redundant points from overlapping scan regions, apply spatial resampling to reduce file size while preserving structural detail, and remove erroneous points introduced by wind-induced branch movement during the multi-position scanning sequence. The original measurement campaign was carried out during relatively calm conditions, however, even minor air movements caused subtle branch motion that was captured as noise in the raw scans. Following this processing workflow, the refined point cloud contained approximately 9 million points representing the isolated tree canopy in clean, representative form. Both the unprocessed raw point cloud and the cleaned, resampled version are provided in LAS 1.4 format in the dataset, allowing users to assess processing decisions and conduct sensitivity analyses if desired. All hardware components, sensors, and their technical characteristics described throughout the methodology are summarized in Table 1.

### Cost analysis of the measurement instruments

To substantiate the “low-cost” designation, the prices of components for custom-built sensors were compared with those of their professional-grade counterparts (Table 2). This comparison does not imply functional equivalence but emphasizes the economic feasibility of high-density spatial sampling. The SN-500 net radiometer measures four-component broadband radiation across the solar and thermal infrared spectra, whereas the BH1750FVI detects only visible illuminance. Deploying an equivalent professional radiometer at all 48 grid positions would have increased radiation-hardware expenditure by a factor of 800, representing a financially prohibitive sum for most research groups. Functional trade-offs can also be observed in the comparison between the FLIR E6 Pro, which provides thermal images of substantially higher spatial resolution than the MLX90640. Similarly, the CR1000 datalogger is equipped with high precision analogue inputs capable of resolving signals on the millivolt scale, which are required for thermal dissipation sap flow probes. This analogue measurement capability cannot be readily replicated with the ESP32 microcontroller at comparable accuracy. Furthermore, professional instruments are delivered as calibrated, ready-to-use systems, whereas low-cost alternatives typically require additional time for circuit assembly, wiring, and firmware development. Consequently, professional instruments were retained as reference standards for broadband radiation and sap flow, while distributed sensing of spatially heterogeneous parameters relied on the low-cost alternatives.

**Table 2:** Quantitative cost comparison between the utilized low-cost sensor components and equivalent professional-grade reference instruments.

Parameter	Low-Cost Component (Unit Price approx.)	Professional Reference (Unit Price approx.)	Cost Reduction Factor
Air Temperature & Relative Humidity	AHT10 (~3 €)	CS215-L (~350 €)	~116
Illuminance / Solar Radiation	BH1750FVI (~5 €)	SN-500 Net Radiometer (~4000 €)	~800
Leaf Surface Temperature	MLX90640 (~80 €)	FLIR E6 Pro (~2000 €)	~25
Data Acquisition	ESP32 (~5 €)	CR1000 Datalogger (~2500 €)	~500

## Data Records

The complete dataset is archived in the open data repository Zenodo (10.5281/zenodo.18039730) under a Creative Commons Attribution 4.0 International License (CC BY 4.0)^18^. All timestamps are recorded in UTC + 1, except for the raw image metadata from the FLIR camera, which are stored in UTC + 2. This offset has already been accounted for in the accompanying data table where the FLIR-derived data are included.

**Fig. 5:**
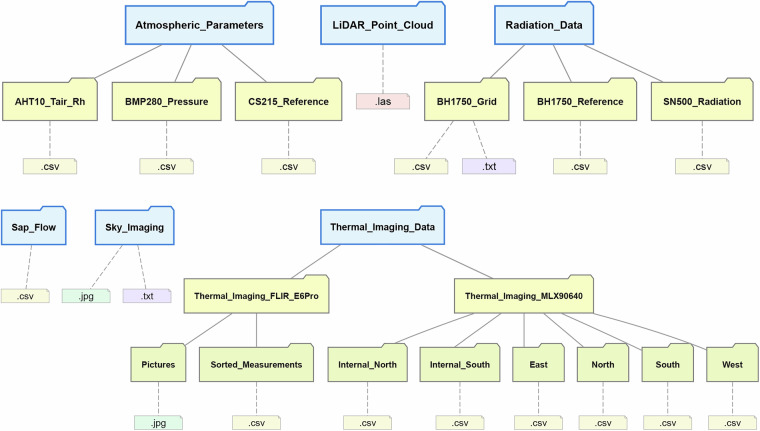
Folder structure of the measurement data.

The dataset comprises six primary data categories with a temporal coverage spanning from 2025-06-11 to 2025-06-14, corresponding to a single clear-sky summer episode, and encompassing atmospheric parameters, radiometric measurements, thermal imaging, sky conditions, sap flow, and three-dimensional point cloud data (Fig. 5, Table 3)^18^. While structurally and functionally rich, the dataset is derived from a single *Tilia cordata* individual and is therefore intended as a structurally explicit benchmark case, rather than a generalized representation of all urban tree species or canopy forms.

**Table 3:** Attribution of measuring instruments, recorded variables, and associated units.

Variable	Unit	Definition	Measurement Method
Air Temperature	°C	Temperature of air at 2 m height in radiation shield	CS215-L, AHT10 sensors
Relative Humidity	%	Relative humidity at 2 m height in radiation shield	CS215-L, AHT10 sensors
Barometric Pressure	hPa	Barometric pressure at 2 m height	BMP280 sensor
SWin	W m^−2^	Incoming shortwave radiation (solar radiation, 385–2105 nm)	SN-500 radiometer
SWout	W m^−2^	Outgoing shortwave radiation (reflected/scattered from ground)	SN-500 radiometer
LWin	W m^−2^	Incoming longwave radiation (thermal radiation from sky/clouds, 5–30 μm)	SN-500 radiometer
LWout	W m^−2^	Outgoing longwave radiation (thermal radiation from ground/grass)	SN-500 radiometer
SWalbedo	%	Albedo	Calculated from SN-500 radiometer
NR	W m^−2^	Net all-wave radiation (SWnet + LWnet)	SN-500 radiometer
Illuminance	W m^−2^	Visible radiation (400–700 nm) quantified as radiometric equivalent	BH1750FVI sensors with hemisphere diffuser
Irradiance	W m^−2^	Visible radiation converted to radiometric equivalent using calibration regression	BH1750FVI + regression
Leaf Surface Temperature	°C	Temperature of outer leaf surface in 8–14 μm thermal band	MLX90640, FLIR E6 Pro thermal sensors
Sap Flux Density	ml cm^−2^ min^−1^	Radial water transport velocity through sapwood	Thermal dissipation probes (Granier method)
Sap Flow	ml min^−1^	Whole-tree transpiration rate, integrated across sapwood area	TDP measurement × sapwood cross-sectional area
ΔT (thermal gradient)	°C	Temperature difference between heated and reference probes	TDP sensors

### Atmospheric parameters

Professional-grade and low-cost air temperature and relative humidity measurements were collected simultaneously in an identical radiation shield at 2 m height. The CS215-L reference sensor (professional-grade instrument) recorded at 5-second intervals and is provided in CSV format with columns for timestamp, battery voltage, panel temperature, air temperature in °C, and relative humidity as a percentage. The AHT10 low-cost sensor (microcontroller-integrated), operated at 20-second intervals, providing concurrent measurements of air temperature and relative humidity. Barometric pressure measurements from a BMP280 sensor, also at 20-second intervals, complete the atmospheric characterization. All three atmospheric datasets maintain temporal coverage >98.9% with no flagged or removed data points.

### Radiation data

The radiometer (SN-500) measurements containing all components of the short- and longwave radiation balance at 5-second intervals are archived as a single CSV file comprising eight radiation variables: incoming shortwave irradiance from the sun and sky (SWin) and outgoing shortwave irradiance reflected or scattered by the surface (SWout), incoming longwave irradiance emitted by the atmosphere and clouds (LWin) and outgoing longwave irradiance emitted by the ground and vegetation (LWout), net shortwave and longwave radiation (SWnet, LWnet), albedo (SWalbedo), and net all-wave radiation (NR). Two illuminance datasets are provided in complementary formats: both a reference BH1750FVI sensor co-located adjacent to the radiometer and a distributed grid of 48 sensors across the measurement domain^18^. For each illuminance source, data are provided in both raw photometric units (lx) and in radiometric units (W m^−2^), with the radiometric conversion derived from the calibration regression described in the Technical Validation. The illuminance grid dataset includes a supporting metadata file specifying the spatial position of each station relative to the tree stem, enabling reconstruction of the two-dimensional radiation heterogeneity beneath the canopy (Fig. 3)^18^. The radiation datasets achieve high temporal completeness, with the SN-500 radiometer providing complete records from 2025-06-12 to 2025-06-14 as well as the reference and grid illuminance sensors maintaining >99.6% data coverage across the measurement period.

### Thermal imaging

Six fixed MLX90640 thermal cameras, positioned at north, east, south, west, and two internal (north and south) locations, recorded leaf surface temperatures at 20-second intervals throughout the entire campaign. Each position yields two complementary data files: an aggregated CSV containing the mean temperature of the central 9-pixel region (which enables direct comparison with the reference FLIR survey data), and a complete raw data file containing all 768 pixel temperatures per measurement interval in calibrated form. The thermal cameras achieved >98.0% data completeness across all positions. An independent FLIR E6 Pro thermal camera was deployed on 2025-06-12 for reference measurements and provides JPG image files with accompanying metadata. A summary CSV file indexes the FLIR measurements and associates each image with its acquisition time and location.

### Sky conditions

A fixed fisheye camera (120° field of view) mounted adjacent to the canopy acquired hemispherical sky images at 15-second intervals from 2025-06-12 05:21 to 2025-06-14 19:05 UTC + 1. The resulting 14,670 JPG images provide a continuous visual record of cloud cover evolution together with the prevailing atmospheric conditions over the measurement period, allowing the application of existing cloud detection algorithms to derive cloud fraction and optical properties.

### Sap flow

Thermal dissipation probes positioned on the north-facing sapwood of the tree recorded radial water transport at 5-second intervals. The sap flow dataset includes raw thermocouple voltage measurements, calculated temperature gradients (ΔT), sap flux density derived via the Granier equation, and integrated whole-tree sap flow scaled to the measured sapwood cross-sectional area (566.44 cm²)^18^. The data completeness exceeds 98.8%, ensuring robust coverage and high data integrity for subsequent analyses.

### Point cloud data

Three-dimensional canopy structure is documented through terrestrial LiDAR scanning, provided in two processing levels. The raw, unfiltered point cloud (LAS 1.4 format, ~29 million points) represents the combined acquisition from three scanning positions without post-processing. A secondary resampled and manually cleaned version (LAS 1.4 format, ~9 million points) contains only points corresponding to the measured tree, with artefacts and background removed.

### Data quality, completeness, and caveats

All datasets exhibit high temporal completeness (>98.0% across all measurement types), with no data points removed or flagged during quality control procedures. Completeness is assessed relative to the operational period of each individual instrument. Measurements of atmospheric parameters, thermal imaging cameras, sap flow, and the illuminance grid were started on June, 11, 2025 at approximately 19:30 UTC + 1, whereas the complete sensor ensemble, including radiation measurements and sky imaging became operational on the early morning of June, 12, 2025, with all devices functioning continuously from this date onwards. Users should note several instrumental characteristics that affect data interpretation. The SN-500 radiometer exhibits thermal offsets during nighttime hours, producing small negative values (<−2 W m^−2^) in the shortwave channel. Measurements below 10 W m^−2^ in the incoming shortwave radiation channel should therefore be interpreted as instrumental artifacts rather than true solar radiation. The BH1750FVI illuminance sensors show increased uncertainty at very low illuminance levels, nighttime readings of zero lux are reliable, but twilight transitions warrant careful evaluation. All thermal measurements assume leaf emissivity of 1.0 where actual values in the 8–14 μm band typically range from 0.96–0.99, and users requiring higher accuracy should apply appropriate corrections^31,32^. Finally, the measurement grid experiences shading from adjacent vegetation (other trees to the north and east, and a sapling to the west), which creates directional shading effects particularly during early and late hours of the day. These effects are partly visible in the radiation grid data and should be considered in spatial analyses.

## Technical Validation

This chapter presents a comprehensive technical validation of the measurement systems and derived data products, demonstrating their accuracy, consistency, and suitability for microclimate research through direct intercomparisons, external references, and physical plausibility checks. Each subsection details the validation procedures and key performance metrics for the individual sensor arrays.

### Air temperature and relative humidity validation

The CS215-L professional sensor and AHT10 low-cost sensor were co-located in the same radiation shield and operated simultaneously throughout the measurement campaign. This parallel deployment enabled direct comparative analyses of measurement accuracy over the three-day period. The comparison of simultaneous measurements across the full deployment yielded detailed statistical insights into the performance characteristics of the low-cost alternative sensors.

**Fig. 6:**
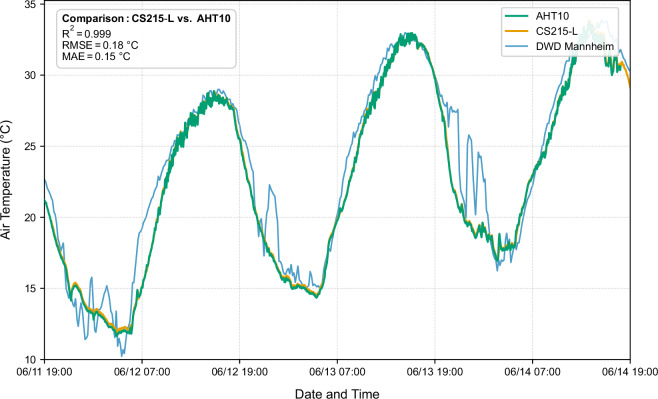
Comparison of the air temperature measured by the two sensors operating on site and the comparative data from the DWD station in Mannheim.

**Fig. 7:**
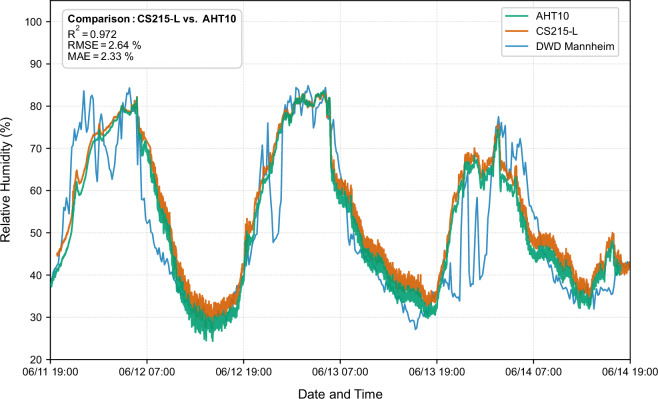
Comparison of the relative humidity of the two sensors operating on site and the comparative data from the DWD station in Mannheim.

For air temperature, the comparison between the two systems revealed exceptional agreement, with a coefficient of determination (R²) of 0.9992, indicating that professional-grade and low-cost sensors captured 99% of the variance in measured temperature variations. The root-mean-square error (RMSE) between systems was only 0.18 K, while the mean absolute error (MAE) measured 0.15 K (Fig. 6). For relative humidity, the agreement remained excellent though marginally lower than for temperature. The R² value of 0.9720 indicates that 97% of variance was captured by the low-cost sensor, with an RMSE of 2.64% and MAE of 2.33% (Fig. 7).

In addition to this co-location comparison, the measurements from both sensors were statistically compared to meteorological data obtained from the German Weather Service (Deutscher Wetterdienst, DWD) station in Mannheim, located approximately 25 km from the measurement site^33^. For air temperature, the CS215-L reference sensor showed an R² of 0.9303 and an RMSE of 1.70 K relative to the DWD measurements, while the AHT10 sensor yielded an R² of 0.9212 and an RMSE of 1.80 K. For relative humidity, the corresponding values were an R² of 0.6964 and an RMSE of 8.65% for the CS215-L and an R² of 0.7461 and an RMSE of 8.31% for the AHT10. As expected, these metrics are less favorable due to the spatial separation and differing local microclimatic conditions between Mannheim and the measurement site in Grünstadt. Nevertheless, the observed temporal trends between the on-site sensors and the DWD reference station closely follow each other, providing an additional indication of the validity and consistency of the collected microclimate data.

These results provide strong evidence that the AHT10 sensor is adequately calibrated and exhibits no significant drift over the measurement period, making it suitable for deployment in distributed sensor networks where cost constraints rather than reference-grade accuracy are the primary limiting factor. The observed differences between systems are entirely attributable to normal sensor-to-sensor manufacturing tolerance and measurement noise. These margins do not meaningfully compromise data utility for microclimate model validation or for characterizing the spatial heterogeneity of environmental conditions beneath tree canopies. The validation thus confirms that resource-constrained research groups can achieve measurement accuracy sufficient for scientific purposes through careful selection and comparison of low-cost sensor alternatives.

### Barometric pressure validation

The BMP280 barometric pressure sensor lacked a co-located professional reference instrument at the measurement site, necessitating validation through comparison with remote reference data. Hourly pressure observations from the DWD station in Mannheim, located approximately 25 km away at 97.95 m elevation, were used for this purpose^34^. However, the Mannheim DWD station is situated 67.05 m lower in elevation than the Grünstadt measurement site (165 m above sea level), creating a systematic pressure difference that required correction before a meaningful comparison can be conducted.

The barometric altitude correction was applied using the exponential barometric formula (Eq. 4):

4 $$p(h)={p}_{0}\cdot \exp \left(-\frac{M\cdot g\cdot \Delta h}{R\cdot T}\right)$$

where ${p}_{0}$ represents the reference pressure at the lower elevation (Mannheim), M is the molar mass of dry air (0.029 kg mol^−1^), g is the gravitational acceleration (9.81 m s^−2^), $\Delta h$ is the elevation difference (67.05 m), R is the universal gas constant (8.314 J mol^−1^ K^−1^), and $T$ is the local air temperature (°C + 273.15) measured on-site^35^. This physically principled correction incorporated temperature-dependent variability in air density, with the correction factor derived from measured temperatures averaging −7.77 hPa but varying on an hourly basis as air temperature changed. By applying the temperature-dependent correction to each DWD measurement, the two datasets were placed on an equivalent basis for direct comparison.

**Fig. 8:**
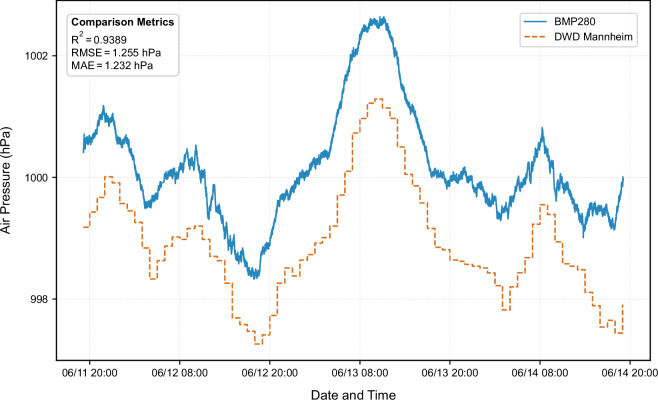
Comparison of barometric pressure between the sensor measured on site and the elevation-adjusted reference data from the DWD station in Mannheim.

Following the application of this altitude correction, the comparison between BMP280 measurements and corrected DWD data revealed high-quality agreement (Fig. 8). The coefficient of determination reached 0.9389, indicating that roughly 94% of the variance in pressure fluctuations was consistently captured. The RMSE between the two measurement systems was 1.25 hPa, while the mean absolute error measured 1.23 hPa. This moderate disagreement, exceeding the BMP280’s laboratory accuracy specification of ±1 hPa, is largely explained by the 25 km horizontal separation between measurement sites and the 1-hour temporal resolution of DWD observations. The high coefficient of determination demonstrates that the BMP280 systematically and accurately tracks pressure variability despite this modest absolute offset, indicating its suitability as a cost-effective sensor for distributed microclimate monitoring.

### Illuminance sensor calibration and validation

Illuminance (lux) and radiometric irradiance (W m^−2^) represent fundamentally different physical quantities requiring careful reconciliation. Lux is a photometric unit that quantifies luminous intensity weighted according to the human eye’s photopic response function (V-lambda curve), which exhibits maximum sensitivity at 555 nm (yellow-green light)^36,37^. In contrast, irradiance is a radiometric unit measuring the complete energy flux of incident solar radiation across the spectrum relevant to photosynthesis and thermal processes, 385–2105 nm for the used pyranometer. The relationship between these quantities is inherently non-constant because the spectral distribution of solar radiation reaching Earth’s surface changes with atmospheric composition and sun angle. This phenomenon is particularly pronounced through Rayleigh scattering, which preferentially removes short wavelengths (blue light) when the solar altitude is low or when clouds and aerosols pervade the atmosphere, thereby changing the apparent luminous efficacy (the ratio lux/irradiance) substantially^38^.

Recognizing this complexity, a calibration procedure was developed to establish an empirical relationship between illuminance and shortwave irradiance for the specific measurement conditions present during the campaign. The reference BH1750FVI sensor positioned adjacent to the SN-500 radiometer was used for this calibration development. Data points where shortwave irradiance was less than 10 W m^−2^ were systematically excluded from the analysis to eliminate nocturnal instrumental artifacts from the radiometer (which exhibits small negative values during nighttime due to thermal gradients in the instrument structure). Following this filtering, a polynomial regression of second order was fitted to the remaining data through least-squares minimization (Eq. 5).

5 $$\mathrm{SWin}\,=\,a\cdot {{\rm{Lux}}}^{2}+b\cdot {\rm{Lux}}+c$$

**Fig. 9:**
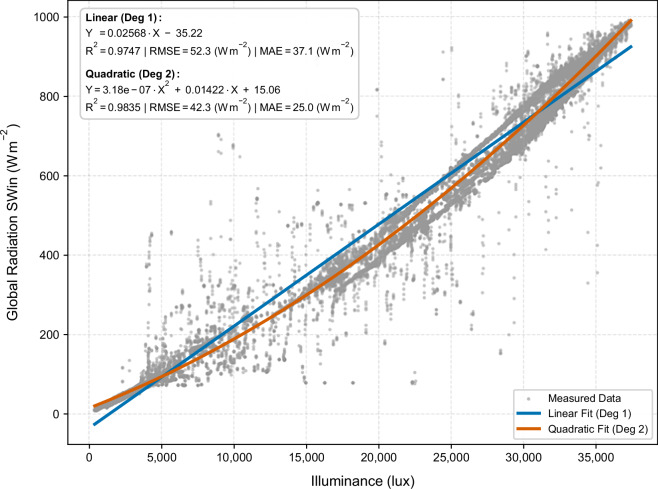
Correlation between illuminance and global radiation using both linear and quadratic regression models.

The superiority of this quadratic formulation relative to a simpler linear model was apparent in the statistical comparison. The quadratic regression ($a=3.46\,\cdot {10}^{-7},{b}=0.01306,{c}=16.39)$ achieved an R^2^ of 0.9888 and an RMSE of 35.7 W m^−2^, whereas the linear model yielded an R^2^ of 0.9786 and an RMSE of 49.3 W m^−2^ (Fig. 9). The improvement in fit quality (ΔR^2^ = 0.0102, ΔRMSE = 13.6 W m^−2^) justifies the additional model complexity and is physically interpretable: the quadratic term directly captures the non-linear response of the relationship to spectral variations. Testing of third-order and higher-order polynomials revealed negligible improvements in fit quality (ΔR² < 0.0001) coupled with substantially increased overfitting risk. Therefore, the second-order polynomial was retained as the optimal calibration model.

To further assess the external consistency of the radiometric measurements, the SN-500 reference radiometer was compared against global radiation data from the DWD station in Mannheim, located approximately 25 km from the measurement site^39^. The DWD data are provided as 10-minute sums of global irradiance in J cm^−2^ and were converted to instantaneous irradiance in W m^−2^ using the irradiance Eq. 6.

6 $$[{\rm{W}}\cdot {{\rm{m}}}^{{-}2}]=\frac{{energy}\,[J{{cm}}^{-2}]\cdot 10000{[{cm}}^{2}{m}^{-2}]}{600[s]}$$

**Fig. 10:**
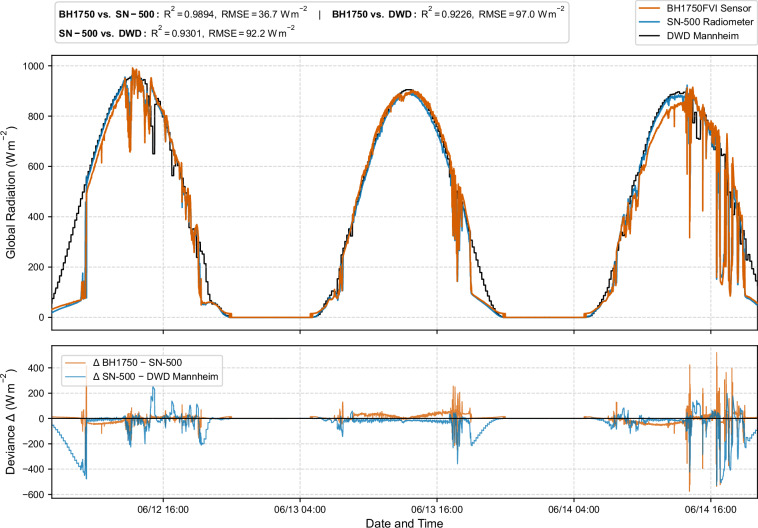
Comparison of global shortwave radiation measured on site with the derived irradiance values and the DWD data from Mannheim.

The comparison between the SN-500 and the converted DWD global radiation yielded an R^2^ of 0.9335 and an RMSE of 92.00 W m^−2^, indicating very good agreement given the spatial separation and differences in local cloud conditions (Fig. 10).

In a second step, the irradiance values derived from the calibrated BH1750FVI illuminance measurements were also evaluated against the DWD station data. Despite the additional uncertainty introduced by the lux-to-irradiance conversion and the distance between Mannheim and the field site, the converted BH1750FVI data still achieved an R² of 0.9253 and an RMSE of 96.97 W m^−2^ relative to the DWD global radiation. The slightly higher errors relative to the direct SN-500-DWD comparison are consistent with the expected propagation of calibration uncertainty and the sensitivity of luminous efficacy to local spectral and cloud conditions. At the same time, the close temporal co-variation between the on-site measurements and the DWD reference confirm that both the pyranometer and the calibrated illuminance sensor reliably capture the diurnal dynamics and synoptic-scale variability of incoming shortwave radiation.

**Fig. 11:**
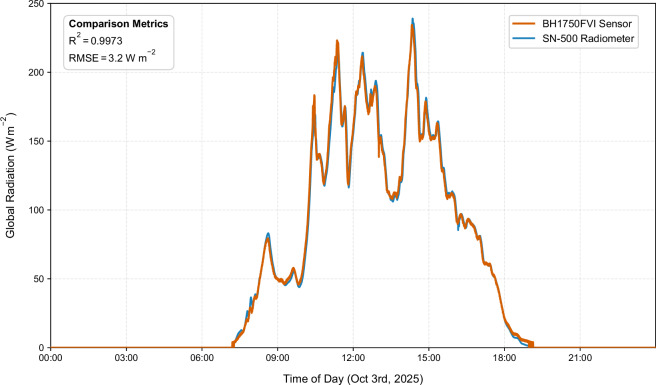
Comparison of global radiation measured in October with derived irradiance values.

However, a critical finding emerged from supplementary measurements conducted approximately four months after the primary measurement campaign. When identical sensor types were deployed, a markedly different calibration curve was obtained despite using the same hardware. The quadratic regression ($a=-4.31\cdot {10}^{-8},b=0.023743,c=3.79)$ results from October yielded an R² of 0.9973 and an RMSE of only 3.23 W m^−2^, representing improvement compared to the June results (RMSE reduction of 32.47 W m^−2^) (Fig. 11). This substantial seasonal variation in calibration efficacy directly reflects changes in atmospheric aerosol optical depth, water vapor content, and ozone absorption between the two measurement periods^40^. The finding underscores a critical methodological principle: luminous efficacy is inherently variable, site-specific, and season-dependent. Conversion of illuminance to radiometric units cannot rely on historical calibrations from different seasons or locations but rather demands contemporaneous radiometer observations during each measurement campaign. The calibration regression derived from the June measurements was therefore applied uniformly to convert all 48 grid station measurements from lux to W m^−2^ for the June campaign, with the explicit caveat that this conversion is valid only for the specific atmospheric conditions present during the measurement period.

### Illuminance grid sensor intercomparison

Implicit in the approach of applying a single calibration regression to all 48 grid sensors is the assumption that these sensors respond identically to both radiation intensity variations and spectral variations. To assess this assumption, a dedicated 27-hour validation study was conducted in which all 48 BH1750FVI sensors were co-located at a single measurement site under natural outdoor conditions. The validation protocol spanned the period from 2025-06-08 17:00 UTC + 2 to 2025-06-09 20:00 UTC + 2, ensuring a complete diurnal cycle including morning and evening low-light conditions as well as high-intensity midday radiation periods. All sensors were configured identically as well as oriented at the same viewing angle, and the experimental setup aimed to expose them to approximately equivalent irradiance levels. However, owing to the open-air conditions and the finite spatial footprint of the 48 sensors, it was not possible to guarantee that each sensor received precisely identical radiation throughout the entire measurement period. Consequently, this intercomparison was principally designed to assess whether all sensors produced generally comparable measurements across the range of ambient radiation conditions. Notably, this period included times of intense solar irradiance as well as rapidly fluctuating radiation intensities induced by transient cloud cover. Such variability ensured that the sensors were tested across all typical radiation scenarios expected during the campaign.

Statistical evaluation of this intercomparison dataset employed the intraclass correlation coefficient (ICC) calculated using a two-way mixed effects model for absolute agreement (Eq. 7):

7 $${\rm{ICC}}=\frac{{\rm{MSR}}-{\rm{MSE}}}{{\rm{MSR}}+(k-1)\cdot {\rm{MSE}}}$$

where MSR represents mean-square reproducibility (variance among measurement times), MSE is the mean-square error, and k is the number of sensors (48 in this case). The ICC result of 0.9801 with 95% confidence interval indicates exceptionally high agreement among sensors and confirms that manufacturing tolerance is negligible relative to measurement sensitivity. This level of agreement provides strong justification for treating all 48 sensors as reliable units directly from the factory, without applying individual calibration offsets.

Supplementary analysis using Bland-Altman methodology quantified systematic bias between each sensor and the group mean. The mean Bland-Altman bias was +95.04 lux, with 95% limits of agreement spanning −1656.02 to +1656.02 lux. This range of ±1656 lux corresponds to approximately ±5.0% of the full measurement range (0–30,664 lux observed during the validation period), which is considered an acceptable tolerance band for the intended application of spatial mapping of radiation heterogeneity. Group-level statistics further reinforced this conclusion: comparison of each sensor against the mean of all 48 sensors yielded a mean R² of 0.9807 and a mean RMSE of 815.33 lux (equivalent to 2.66% of the mean signal amplitude). These findings provide strong empirical evidence that the 48 sensors demonstrate adequate consistency and that the single calibration regression can be reliably applied to all grid positions without introducing unacceptable errors. The internal redundancy evident in these statistics provides confidence in the spatial patterns documented in the grid measurements and supports the validity of high-resolution spatial radiation mapping achieved through the distributed sensor network.

Additionally, it is worth noting that during nighttime periods, all sensors registered readings of zero lux, in line with expected darkness conditions and confirming sensor responsiveness over the full illumination range.

### Thermal imaging sensor calibration

The six MLX90640 thermal cameras were subjected to a cross-calibration procedure to establish consistency across all measurement positions and account for manufacturing variation in factory calibration. The calibration was conducted using a 27-hour controlled exposure during which all thermal cameras were simultaneously aimed at an identical measurement target. All cameras were mounted on a custom 3D-printed fixture positioned identically relative to the target to minimize geometric variation between viewing positions. By exposing all sensors to the same radiant field over an extended period encompassing significant temperature variation throughout a full diurnal cycle, the calibration process captured sensor performance under realistic conditions rather than at a single fixed temperature.

**Fig. 12:**
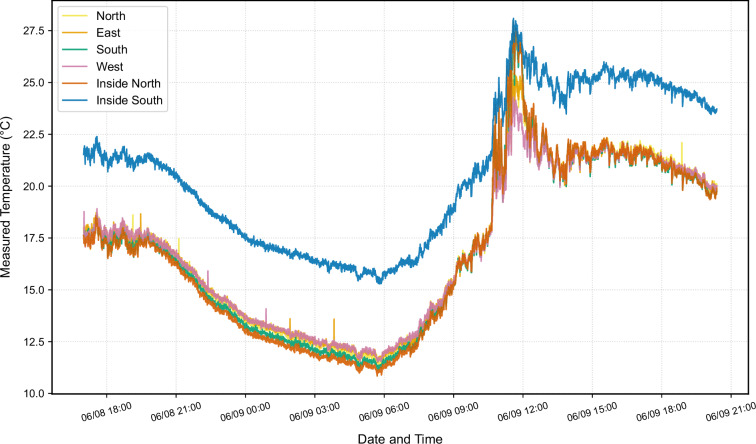
Comparison of the measured temperatures when viewing the same surface with the six thermal cameras.

Comparison of measurements during this calibration period revealed that five of the six cameras operated within acceptable factory calibration tolerance, but station “Inside_South” exhibited a substantial systematic offset of +3.787 °C relative to the mean of the remaining cameras (Fig. 12). This offset magnitude substantially exceeds acceptable tolerance limits (±1 °C) and warranted correction. Accordingly, individual correction offsets were applied to each station based on their deviation from the group mean: station “South” (+0.074 °C), station “North” (−0.079 °C), station “West” (−0.053 °C), station “East” (−0.070 °C), station “Inside_South” (−3.787 °C), and station “Inside_North” (+0.128 °C). Given the experimental setup, where the captured surface area and thus the radiant temperature field were assumed to be highly similar across all sensors, these corrections were deemed necessary, particularly for station “Inside_South”, which fell outside the predefined acceptable deviation range.

After applying these appropriate correction offsets, the post-calibration statistics demonstrated the desired performance: the intraclass correlation coefficient reached 0.9990, the Bland-Altman bias (versus the group mean) fell below 0.01 °C for all stations, the mean R² for pairwise camera comparisons was 0.9955, and the mean RMSE among cameras was only 0.25 °C. These metrics confirm that all six thermal cameras now operate with excellent mutual agreement, providing confidence in spatial comparisons of leaf surface temperatures across the measurement domain.

**Fig. 13:**
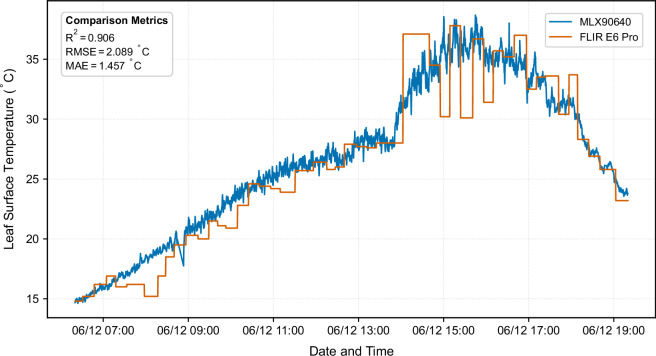
Comparison of the measured leaf surface temperatures in the western direction of the MLX90640 sensor and the measurements of the FLIR thermal imaging camera.

The FLIR E6 Pro thermal camera used for supplementary measurements was factory-calibrated by FLIR Systems on 22 December 2024 at their calibration facility in Estonia, and is specified to have an accuracy of ±2 °C. In addition to the manufacturer calibration, an independent external calibration check was performed by a certified calibration service (TOPA Kalibrierservice, Hohenpeißenberg, Germany), which confirmed the instrument performance within the stated accuracy range. On this basis, the camera is considered to provide valid reference measurements for the evaluation of the MLX90640 thermal cameras, and no further adjustments were applied to its calibration. For the comparison against the corrected MLX90640 cameras (conducted during the temporal window of overlap from 06:20 to 19:20 UTC + 1 on 2025-06-12), measurements from the FLIR E6 Pro were focused on a small central region of the image. To ensure methodological consistency, the MLX90640 data were similarly processed by computing the mean value of the central 9 pixels (corresponding to the FLIR field of view) for each comparison. Across all measurement stations, this analysis yielded satisfactory R² values ranging from 0.803 to 0.906 and low RMSE values between 1.29 K and 2.49 K (Fig. 13). These performance metrics are consistent with the stated accuracy specifications of both instrument types and would be expected to improve further if the temporal resolution of the FLIR measurements matched the ones of the continuously recording MLX90640 cameras. These results confirm the suitability of the DIY thermal camera system for generating data appropriate for subsequent comparative analyses.

### Sap Flow Measurements: Validation and Physical Consistency

The thermal dissipation probe (TDP) system operated reliably without malfunction throughout the entire 3-day measurement period, maintaining stable thermal excitation of the heated probe and continuous acquisition of temperature differentials between heating and reference probes.

Integration of the measured sap flux density over the estimated sapwood area and across the 3-day measurement period yielded peak daily transpiration of approximately 65.48 liter on 2025-06-12, a clear-sky day with maximal radiation receipt. This estimated transpiration rate is physically plausible for a 30-year-old *Tilia cordata* specimen of the measured dimensions and is consistent with independent literature estimates^29,30^. Ahongshangbam *et al*.^41^ reported comparable transpiration rates for urban linden trees measured under hot, clear-sky conditions in Nordic climate settings, providing external validation of the present measurements. These agreements with published values across different geographic contexts provide strong confidence in the quality and realism of the sap flow measurements.

### Three-Dimensional Canopy Structure: Point Cloud Quality

Terrestrial laser scanning and subsequent data processing yielded high-quality three-dimensional characterization of the tree canopy structure. The three independent Topcon GLS-1500 scans were successfully merged without gap artifacts or discontinuities, indicating that an adequate overlap existed between adjacent scan positions to ensure seamless integration. Manual cleaning and resampling using Topcon ScanMaster software reduced the dataset to approximately 9 million points representing the tree canopy in clean, representative form. This data reduction was accomplished through spatial resampling and manual removal of erroneous points while preserving the essential geometric structure. The processed point cloud was validated through comparison with field measurements collected during tree characterization.

Quantitative analysis of the cleaned point cloud confirmed excellent agreement with field measurements. The total tree height derived from the point cloud was 9.64 m (matching the direct measurement), canopy diameter in the east-west direction was 10.93 m, canopy diameter in the north-south direction was 10.61 m, and Leaf Area Index (LAI) calculated from point density metrics was 4.4 m² m^−2^. This LAI value is consistent with literature values for mature *Tilia cordata*, which typically range from 4.0 to 5.5 m² m^−2^. Leaf Area Density (LAD) calculated from a dense leaf cluster on the north side of the canopy was 2.04 m^2^ m^−3^. These corroborating measurements from multiple independent approaches provide strong confidence in the quality of the three-dimensional reconstruction and support its suitability for parameterization of microclimate simulation models.

### Uncertainty analysis

While the BH1750FVI datasheet specifies a potential manufacturing tolerance of up to ±20%, the empirical intercomparison of the 48 sensors used in this study (Illuminance Grid Sensor Intercomparison section) demonstrated a higher precision with a mean bias of only 95.04 lux and limits of agreement of ±5.0%. Consequently, for the uncertainty quantification of the derived irradiance (SWin), we used this empirical value of ${\sigma }_{{Lux}}$ = 5% for Gaussian error propagation. This approach constrains the total uncertainty of the W m^−2^ values to a range that reflects the actual performance of the deployed sensor batch, rather than a theoretical worst-case manufacturer specification.

The resulting total uncertainty (${\sigma }_{{SWin}}$) follows a characteristic pattern common in optoelectronic measurements. During low-light conditions, the relative error can appear high (up to 39.93% at 90 W m^−2^) while the absolute deviation remains negligible. In contrast, during peak midday radiation, the absolute uncertainty increases (±63.54 W m^−2^ at 742.20 W m^−2^) while the relative error drops significantly to 8.56%. This distribution of uncertainty confirms that while measurement noise exists, absolute accuracy remains robust for characterizing the spatial heterogeneity of the urban microclimate.

The quadratic regression function from the Illuminance Sensor Calibration and Validation section, used to convert illuminance (lx) to global irradiance (W m^−2^), is specifically applicable to the atmospheric conditions of the June measurement period. A comparison with identical sensors deployed in October (Fig. 11) indicates that seasonal changes in sunlight’s spectral composition, resulting from variations in aerosol optical depth, water vapor, and solar zenith angle, lead to a significantly different calibration curve (RMSE = 3.23 W m^−2^ in October versus 35.7 W m^−2^ in June). Therefore, users must utilize contemporaneous pyranometer reference data for accurate conversion of illuminance to radiometric units. Applying the June calibration function to other seasons or locations can result in systematic biases whose magnitude depends on the prevailing atmospheric conditions. The available two-season comparison does not support the derivation of a generalized seasonal correction factor.

Regarding the sap flow measurements, a sensitivity analysis revealed that the determination of $\Delta {T}_{\max }$ is a critical factor. A minor variation of ±2% in the $\Delta {T}_{\max }$ baseline results in a mean change of 16.33% in the calculated daily sap flow sum. While this sensitivity highlights the challenges of absolute quantification using the Granier method, it does not affect the observed diurnal rhythms or the relative response of the tree to microclimatic fluctuations.

Beyond the thermal signal processing, the total volumetric sap flow (F) scales linearly with the estimated sapwood area $({A}_{s})$. To quantify the impact of potential errors in the allometric estimation of ${A}_{s}$, a sensitivity test was conducted. A variation of ±5% in the sapwood area resulted in a proportional ±5% change in the daily transpiration sum (57.51 l baseline vs. 60.38 l and 54.63 l respectively). This linear dependency implies that any bias or uncertainty in the allometric sapwood-area estimate directly translates into an equivalent relative uncertainty in the absolute water-use quantification and should therefore be treated as a systematic component of the overall error budget.

All thermal camera measurements were recorded using the same emissivity setting (ε = 1.0). Broadleaf vegetation, however, typically exhibits emissivity values between 0.96 and 0.99 in the 8–14 µm spectral band^31,32^. Assuming a perfect blackbody therefore leads to a systematic underestimation of true leaf surface temperature. Using the Stefan-Boltzmann law a measured radiant temperature of 35 °C with a realistic leaf emissivity of ε = 0.96 corresponds to a true leaf temperature that is 3.16 K higher when corrected. Users requiring absolute thermodynamic accuracy for leaf energy balance calculations should therefore apply an emissivity correction to the raw pixel values provided in the dataset. For relative comparisons across camera positions or over the diurnal cycle, the measurements remain fully comparable, as all cameras were operated with identical emissivity settings.

## Data Availability

All data are archived and openly available in the Zenodo repository (10.5281/zenodo.18039730). For technical questions regarding data access or formatting please contact Peer Schöneberger (p.schoeneberger@geo.uni-mainz.de).

## Code availability

No code is necessary to access or analyze the dataset.

## References

[1] Simon, H. *et al*. Modeling transpiration and leaf temperature of urban trees – A case study evaluating the microclimate model ENVI-met against measurement data. *Landscape and Urban Planning* **174**, 33–40 (2018).

[2] Eingrüber, N., Korres, W. & Schneider, K. Microclimatic field measurements to support microclimatological modelling with ENVI-met for an urban study area in Cologne. *Adv. Sci. Res.* **19**, 81–90 (2022).

[3] Mühlbauer, L. K. *et al*. Arduinos in the wild: A novel, low‐cost sensor network for high‐resolution microclimate monitoring in remote ecosystems. *Ecol Sol and Evidence* **4**, e12255 (2023).

[4] Nava-Pintor, J. A. *et al*. Development and Evaluation of Solar Radiation Sensor Using Cost-Effective Light Sensors and Machine Learning Techniques. *Technologies* **13**, 182 (2025).

[5] Mao, F., Khamis, K., Krause, S., Clark, J. & Hannah, D. M. Low-Cost Environmental Sensor Networks: Recent Advances and Future Directions. *Front. Earth Sci.* **7**, 221 (2019).

[6] Newman, A. J. *et al*. The High-resolution Urban Meteorology for Impacts Dataset (HUMID) daily for the Conterminous United States. *Sci. Data* **11**, 1321 (2024). 39632886 10.1038/s41597-024-04086-2 PMC11618800

[7] Wang, A. *et al*. Hyperlocal environmental data with a mobile platform in urban environments. *Sci. Data* **10**, 524 (2023). 37543703 10.1038/s41597-023-02425-3 PMC10404226

[8] Bosch, M. *et al*. Evaluating urban greening scenarios for urban heat mitigation: a spatially explicit approach. *R. Soc. open sci.* **8**, 202174 (2021). 34909207 10.1098/rsos.202174 PMC8652265

[9] Calhoun, Z. D. *et al*. Estimating the effects of vegetation and increased albedo on the urban heat island effect with spatial causal inference. *Sci Rep* **14**, 540 (2024). 38177220 10.1038/s41598-023-50981-w PMC10766998

[10] Schöneberger, P. *et al*. Enhancing urban microclimate simulations: Validating ENVI-met’s accuracy in modeling multi-directional radiative fluxes and mean radiant temperature in subtropical hong kong. *Building and Environment* **284**, 113475 (2025).

[11] Qin, H. & Zhou, B. Optimizing Vegetation Configurations for Seasonal Thermal Comfort in Campus Courtyards: An ENVI-Met Study in Hot Summer and Cold Winter Climates. *Plants* **14**, 1670 (2025). 40508344 10.3390/plants14111670 PMC12157141

[12] Yazdi, H., Shu, Q., Rötzer, T., Petzold, F. & Ludwig, F. A multilayered urban tree dataset of point clouds, quantitative structure and graph models. *Sci. Data* **11**, 28 (2024). 38177188 10.1038/s41597-023-02873-x PMC10767077

[13] Corro, L. M. *et al*. An enhanced national-scale urban tree canopy cover dataset for the United States. *Sci. Data* **12**, 490 (2025). 40128215 10.1038/s41597-025-04816-0 PMC11933301

[14] Richter, R. Forest canopy microclimate regulation during the drought year 2018 at the Leipzig Canopy Crane site. 3 datasets Preprint at, 10.1594/PANGAEA.944139 (2022).

[15] Richter, R. *et al*. Tree species matter for forest microclimate regulation during the drought year 2018: disentangling environmental drivers and biotic drivers. *Sci Rep* **12**, 17559 (2022). 10.1038/s41598-022-22582-6 PMC9584904 36266469

[16] Yi, K. *et al*. High Heterogeneity in Canopy Temperature Among Co‐occurring Tree Species in a Temperate Forest. *JGR Biogeosciences* **125**, e2020JG005892 (2020).

[17] Martynova, M., Sultanova, R., Odintsov, G., Sazgutdinova, R. & Khanova, E. Growth of *Tilia cordata* Mill. in Urban Forests. *SEEFOR* **11**, 51–59 (2020).

[18] Schöneberger, P., Sinsel, T., Simon, H. & Bruse, M. High-resolution multimodal microclimate dataset: thermal, radiative and physiological measurements of a mature linden tree (tilia cordata). *Zenodo*10.5281/ZENODO.18039731 (2025).

[19] Moser-Reischl, A. *et al*. Spatial and temporal dynamics of the leaf area index (LAI) of selected tree species in urban environments. *Urban Forestry & Urban Greening* **107**, 128795 (2025).

[20] Anys, M. & Weiler, M. Rainfall interception by urban trees: Event characteristics and tree morphological traits. *Hydrological Processes* **38**, e15146 (2024).

[21] Campbell Scientific Inc. Campbell Scientific CS215-L Digital Air Temperature and Relative Humidity Sensor (2020).

[22] Aosong Electronic Co. LTD. ASAIR AHT10 Technical Manual (2018).

[23] Bosch Sensortec GmbH. Bosch BMP280 Digital Pressure Sensor Data Sheet V.1.26 (2021).

[24] Apogee Instruments Inc. SN-500-SS & SN-522-SS Net Radiometer (2022).

[25] ROHM Semiconductor Co. Ltd. Ambient Light Sensor IC Series BH1750FVI Digital 16bit Serial Output Type Ambient Light Sensor IC (2011).

[26] Melexis N.V. MLX90640 32x24 IR array Datasheet (2019).

[27] FLIR Systems Inc. FLIR Ex Pro-Series Infrared Cameras with Ignite Cloud (2023).

[28] Granier, A. Evaluation of transpiration in a Douglas-fir stand by means of sap flow measurements. *Tree Physiology* **3**, 309–320 (1987). 14975915 10.1093/treephys/3.4.309

[29] Rahman, M. A., Moser, A., Rötzer, T. & Pauleit, S. Microclimatic differences and their influence on transpirational cooling of Tilia cordata in two contrasting street canyons in Munich, Germany. *Agricultural and Forest Meteorology* **232**, 443–456 (2017).

[30] Gebauer, T., Horna, V. & Leuschner, C. Variability in radial sap flux density patterns and sapwood area among seven co-occurring temperate broad-leaved tree species. *Tree Physiology* **28**, 1821–1830 (2008). 19193565 10.1093/treephys/28.12.1821

[31] Tiralla, N. *et al*. Quantification Of Leaf Emissivities Of Forest Species: Effects On Modelled Energy And Matter Fluxes In Forest Ecosystems. *GES* **12**, 245–258 (2019).

[32] Ribeiro, D., Luz, B. & Crowley, J. K. Spectral reflectance and emissivity features of broad leaf plants: Prospects for remote sensing in the thermal infrared (8.0–14.0 μm). *Remote Sensing of Environment* **109**, 393–405 (2007).

[33] DWD Climate Data Center (CDC). Hourly station observations of 2 m air temperature and humidity for Germany, Version v24.03. (2025).

[34] DWD Climate Data Center (CDC). Hourly station observations of pressure for Germany, Version v24.03 (2025).

[35] Lente, G. & Ősz, K. Barometric formulas: various derivations and comparisons to environmentally relevant observations. *ChemTexts* **6**, 13 (2020).

[36] Dirnberger, D., Reise, C., Heydenreich, W. & Reich, N. H. Simplifying Measurements of Spectral Irradiance. *26th European Photovoltaic Solar Energy Conference and Exhibition*; *3454-3457*, 4, 10.4229/26THEUPVSEC.2011-4AV.1.51 (2011).

[37] Michael, P. R., Johnston, D. E. & Moreno, W. A conversion guide: solar irradiance and lux illuminance. *J. meas. eng.* **8**, 153–166 (2020).

[38] Di Laccio, J. L., Russo, P., Monetta, A., Alonso-Suárez, R. & Abal, G. Atmospheric dependence of the direct, diffuse, and global clear-sky conversion ratios between solar photosynthetic active irradiance and photon flux. *Agricultural and Forest Meteorology* **345**, 109832 (2024).

[39] DWD Climate Data Center (CDC). Hourly station observations of solar incoming (total/diffuse) and longwave downward radiation for Germany, Version v24.03 (2025).

[40] Gröbner, J. *et al*. Spectral aerosol optical depth from SI-traceable spectral solar irradiance measurements. *Atmos. Meas. Tech.* **16**, 4667–4680 (2023).

[41] Ahongshangbam, J. *et al*. Sap flow and leaf gas exchange response to a drought and heatwave in urban green spaces in a Nordic city. *Biogeosciences* **20**, 4455–4475 (2023).

